# Osteosarcoma Metastasis to the Thorax: A Pictorial Review of Chest Computed Tomography Findings

**DOI:** 10.3390/diagnostics14182085

**Published:** 2024-09-20

**Authors:** Khalid Abdulaziz Alduraibi, Jawaher Ali Towhari, Hatim Abdullah Alebdi, Bader Zaid Alfadhel, Ghazi S. Alotaibi, Subha Ghosh, Mnahi Bin Saeedan

**Affiliations:** 1 Department of Radiology, King Faisal Specialist Hospital and Research Center, Riyadh 11533, Saudi Arabia; khalid.alduraibi@gmail.com (K.A.A.); jawaher.towhari@hotmail.com (J.A.T.); hatim.alebdi@gmail.com (H.A.A.); dr.baderalfadhel@gmail.com (B.Z.A.); 2 Division of Hematology, Department of Medicine, College of Medicine, King Saud University, Riyadh 11362, Saudi Arabia; galo@ksu.edu.sa; 3 Department of Radiology, Thoracic Imaging, Cleveland Clinic, Cleveland, OH 44195, USA; ghoshs2@ccf.org

**Keywords:** osteosarcoma, lung metastasis, chest computed tomography, unusual thoracic metastasis

## Abstract

**Background:** Osteosarcoma, a primary bone malignancy in children and adolescents, frequently metastasizes to the lungs, contributing significantly to morbidity and mortality. **Lung Metastases:** At diagnosis, 15–20% of patients present with detectable lung metastases. Chest computed tomography (CT) is vital for the early detection and monitoring of these metastases. Lung involvement typically presents as multiple nodules of varying sizes and can include atypical features such as cavitation, cystic lesions, ground-glass halos, intravascular tumor thrombi, and endobronchial disease. **Additional Findings:** Pleural metastasis often occurs alongside pulmonary disease, and complications like spontaneous pneumothorax may arise. Additional findings may include thoracic lymphadenopathy, cardiac tumor thrombus, and chest wall deposits. **Conclusion:** Familiarity with these imaging patterns is essential for radiologists to ensure timely diagnosis and effective management. This review highlights the critical role of chest CT in detecting and characterizing osteosarcoma metastasis.

## 1. Introduction

Osteosarcoma is the most common primary bone malignancy in children and adolescents [1]. At diagnosis, metastasis is detected in 15%–20% of cases, although nearly all patients are presumed to have subclinical micrometastatic disease [2,3,4]. Metastatic disease significantly contributes to the poor prognosis of osteosarcoma, with a survival rate of around 20% in patients with metastasis [5,6,7], compared to a 60% to 70% 5-year survival rate in patients with localized disease, largely due to the introduction of systemic chemotherapy [8].

Metastasis from osteosarcoma primarily spreads hematogenously, with the lungs being the most common site of involvement. Computed tomography (CT) is the preferred imaging modality for detecting pulmonary metastases. Osteosarcoma metastasis in the chest can present with both typical and atypical imaging features and may also affect non-pulmonary thoracic structures [9,10,11,12].

This review aims to illustrate and discuss the common and uncommon sites and imaging features of metastatic osteosarcoma in the chest, emphasizing the critical role of CT in initial staging and follow-up.

## 2. The Lungs

Lung metastases exhibit a wide range of appearances on CT, underscoring the importance of recognizing their diverse manifestations. While metastatic lesions primarily affect the lung parenchyma, they can also involve the lung vasculature, bronchial tree, and lymphatic system. Parenchymal metastases often present as solid nodules. However, atypical presentations are not uncommon and may include consolidations, nodules with ground-glass halos, cavitations, and cystic lesions.

### 2.1. Parenchymal Metastasis

**Figure 1:**
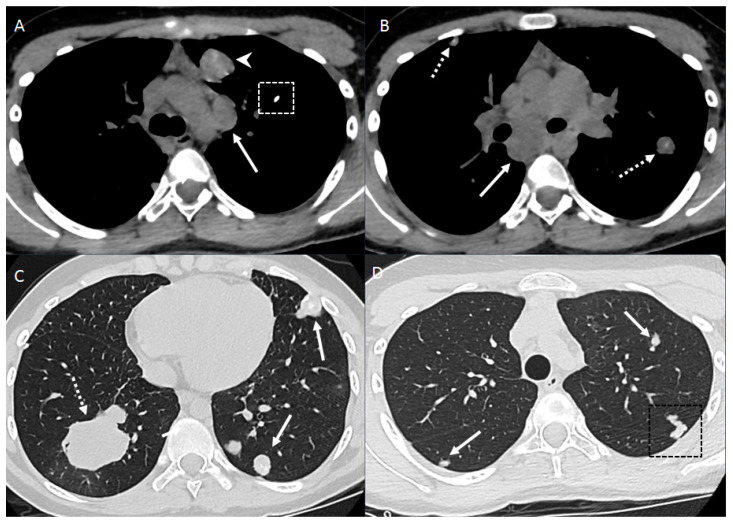
Patient with osteosarcoma lung metastasis and metastatic mediastinal lymphadenopathy. Axial non-enhanced images (**A**,**B**) show enlarged para-aortic (arrow in image **A**) and subcarinal (arrow in image **B**) lymph nodes, left upper lobe lung mass with heterogeneous intrinsic calcifications (arrowhead), and lung nodules with central calcification (dashed arrows) and diffuse calcifications (dotted box). Axial non-enhanced images (**C**,**D**) show right lower lobe lung mass (dashed arrow), bilateral lung nodules of variable size and some nodules associated with central calcifications lung nodules (arrows) and left upper lobe peripheral branching opacity reflecting intravascular metastasis (dotted box).

Pulmonary metastases from osteosarcoma typically present as well-defined, rounded, solid nodules. The multiplicity of lung nodules, especially when numbering greater than seven, and sizes exceeding 5 mm are highly suggestive of metastatic disease, with specificity increasing with larger nodules (>6 mm specificity, ~90%; >13 mm specificity, 100%) [13]. Approximately 60% of these nodules may demonstrate calcification, which is significantly higher compared to the 12% observed in benign nodules [13] (Figure 1). Interval changes in nodule number or size following chemotherapy further support the diagnosis of metastasis [13].

A retrospective analysis that compared CT findings and pathology reports of resected nodules reported that about 14% of osteosarcoma lung metastases were not nodular and could appear as consolidations, cavitation, ground-glass halo, and striae. Striae is a potential pitfall and could be interpreted as linear atelectasis or fibrosis [10].

**Figure 2:**
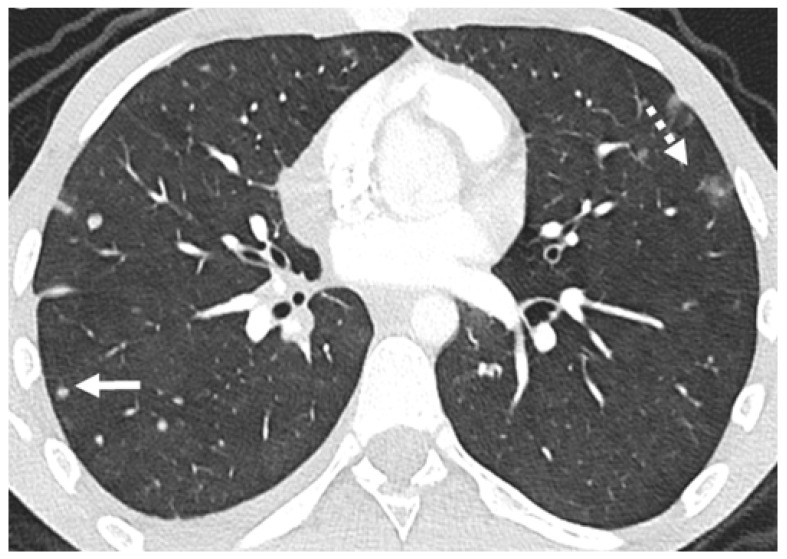
Patient with osteosarcoma and lung metastasis. Axial enhanced image shows multiple lung nodules, some of them subpleural on their location (arrow), and some demonstrate surrounding ground-glass opacities, illustrating the “halo sign” (dashed arrow).

Hemorrhagic metastases, characterized by nodular opacities with a ground-glass halo (Figure 2), are another distinct pattern resulting from neovascular tissue fragility [14].

**Figure 3:**
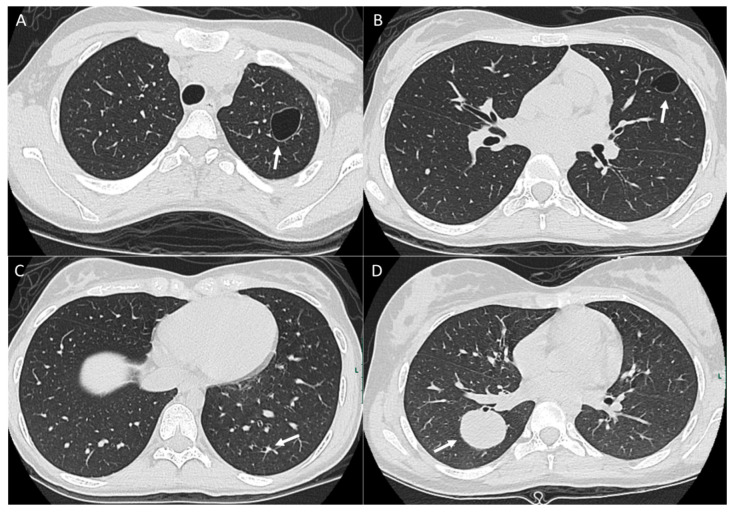
Patient with osteosarcoma and lung metastasis. Axial non-enhanced images (**A**,**B**) show cystic lesions in the left upper lobe (arrows). Axial non-enhanced image (**C**) shows a left lower lobe small cystic lesion with a nodular component (arrow). These cystic lesions were new compared to baseline CT and compatible with cystic metastasis. Axial non-enhanced image (**D**) from follow-up CT shows interval development of a large right lower lobe metastatic lung mass (arrow).

**Figure 4:**
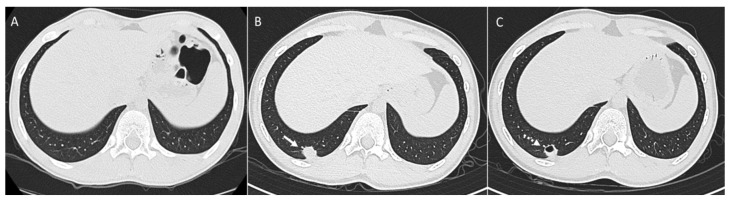
Patient with osteosarcoma and lung metastasis. Axial non-enhanced image (**A**) shows no lung nodule. Axial non-enhanced image (**B**) from a follow-up CT shows an interval development of a right lower lobe metastatic lung nodule (arrow). Axial non-enhanced image (**C**) from a subsequent follow-up CT shows an interval development of cavitation (dashed arrow).

Cystic metastatic lesions have been described (Figure 3), where initially solid nodules evolve into cystic formations due to progressive distension and rupture of alveolar air sacs from bronchiolar infiltration and a check-valve mechanism [14,15,16]. Cavitation within lung metastases, often resulting from chemotherapy-induced tumor necrosis, is a noteworthy feature [14,17] (Figure 4).

### 2.2. Vascular Metastasis

**Figure 5:**
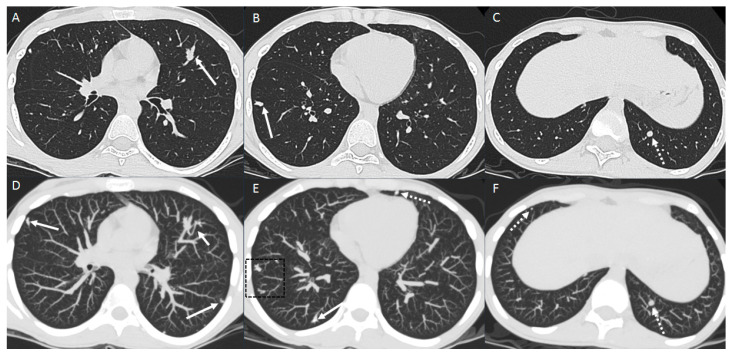
Patient with osteosarcoma and lung metastasis. Axial non-enhanced images (**A**–**C**) with corresponding axial maximum-intensity projection images. (**D**–**F**) show multiple bilateral tubular opacities with a branching pattern (arrows) and vascular tree in bud appearance (dashed box in image **E**), which are compatible with intravascular metastasis compared to multiple rounded nodules (dashed arrows).

**Figure 6:**
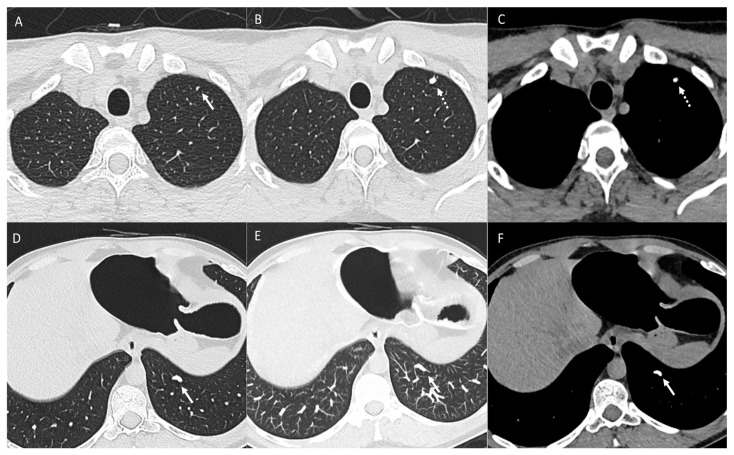
Patient with osteosarcoma and lung metastasis. Axial non-enhanced image (**A**) shows a small left upper lobe nodule (arrow). Axial non-enhanced images in the lung and mediastinal windows (**B**,**C**) from follow-up CT show an interval increase in size with new diffuse calcification (dashed arrows). Axial non-enhanced image (**D**) and corresponding axial maximum-intensity projection image (**E**) and axial mediastinal window (**F**) show a branching calcified lung nodule, which is compatible with calcified intravascular metastasis (arrows).

Pulmonary vascular metastasis is increasingly recognized due to the availability of CT thin slices [12,18,19]. It is characterized on CT by a dilated, non-tapering distal pulmonary artery, which may show interval mineralization or ossification over time [18]. Radiologists should consider vascular metastasis when encountering multifocal tubular peribronchovascular branching opacities with adjacent patent airways [12,18,19] (Figure 1, Figure 5 and Figure 6).

### 2.3. Endobronchial Metastasis

**Figure 7:**
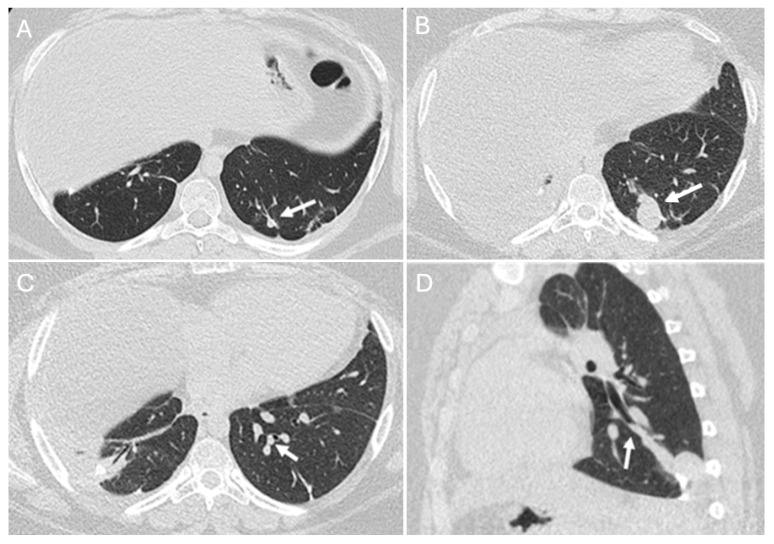
Patient with osteosarcoma and recurrent lung metastasis post metastasectomy and endobronchial metastasis. Axial non-enhanced image (**A**) shows a small left lower lobe nodule along the surgical line (arrow), suggesting recurrent disease. Axial non-enhanced image (**B**) from a follow-up CT shows an interval increase in size (arrow) representing disease recurrence. Axial (**C**) and sagittal (**D**) images from the follow-up CT show an interval development of endobronchial extension (arrows).

Endobronchial metastasis, though rare, can arise from various mechanisms, including hematogenous spread to the bronchus, direct invasion from a parenchymal lesion, or extension from metastatic mediastinal or hilar lymphadenopathy (Figure 7) [20]. Endobronchial metastasis can present as obstructive lesions leading to post-obstructive pneumonia or atelectasis [20,21,22].

### 2.4. Lymphangitic Carcinomatosis

Lymphangitic carcinomatosis, though infrequent in osteosarcoma, can present with nodular peribronchovascular, interlobular, and fissural thickening, often accompanied by lymphadenopathy [12,23].

### 2.5. Management

**Figure 8:**
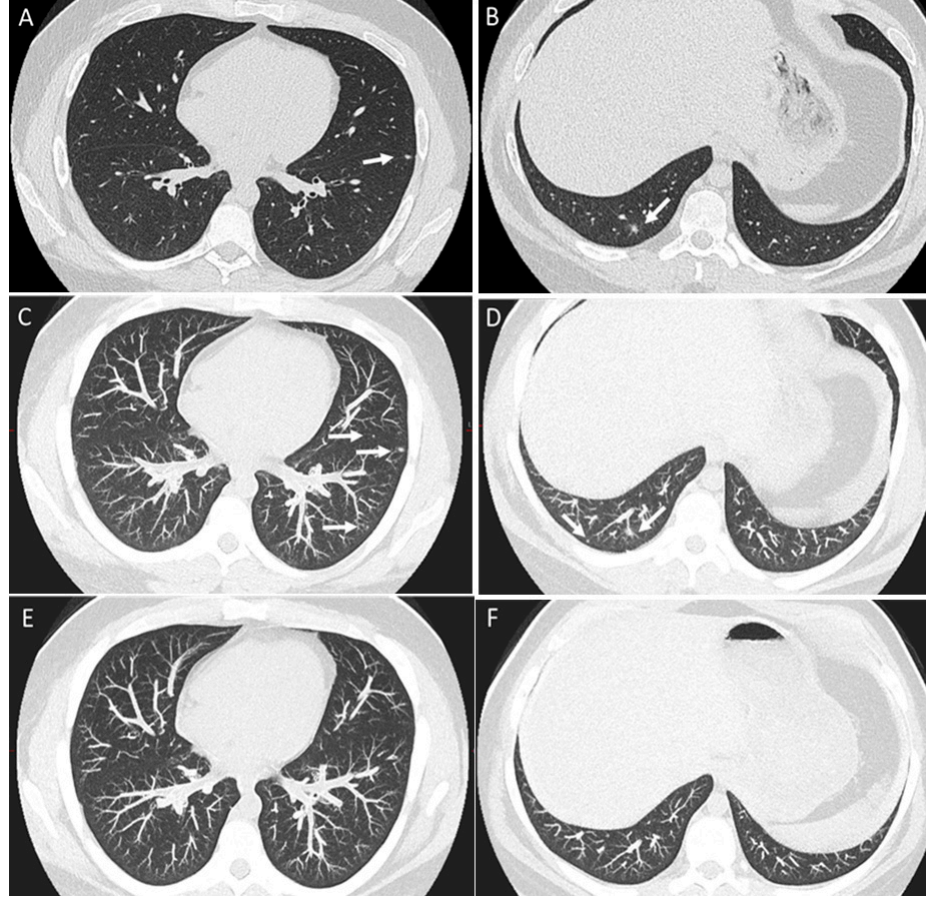
Patient with osteosarcoma and indeterminate pulmonary nodules. Axial non-enhanced images (**A**,**B**) and corresponding maximum-intensity projection images (**C**,**D**) show newly developed pulmonary nodules (arrows) for which short-term follow-up was recommended. Axial non-enhanced images using maximum-intensity projection (**E**,**F**) from a follow-up CT after three weeks show interval resolution of these nodules, which are compatible with resolved infection/inflammatory process.

False-positive findings on chest CT can pose significant challenges, often arising from benign entities such as infection, granulomas, post-inflammatory scarring, or atelectasis, which can mimic metastatic nodules (Figure 8) [10]. To mitigate these issues, serial imaging to assess interval changes and a thorough review of the patient’s clinical history are essential.

Indeterminate pulmonary nodules represent a diagnostic challenge, especially when the nodules are less than 5 mm in diameter. These nodules often require close follow-up with interval imaging to monitor for growth or changes in characteristics. There are no significant differences in outcomes between patients without pulmonary nodules and those with a single nodule smaller than 5 mm. Furthermore, the number of pulmonary nodules detected on thin-slice CT scans at disease onset may hold greater prognostic significance than their size [24]. This observation supports the Children’s Oncology Group criteria for defining metastatic disease, which are based on the presence of either a single nodule larger than 10 mm or more than three nodules greater than 5 mm [25].

Imaging with (18)F-fluorodeoxyglucose positron emission tomography [(18)F-FDG PET] offers advantages over traditional CT in identifying metabolically active cancerous tissue. However, CT remains superior for detecting small nodules and providing a detailed evaluation of the lung parenchyma. While PET-CT can enhance the specificity of diagnosing metastasis, especially in equivocal cases, its role is complementary to CT, particularly in preoperative planning and assessing treatment response. The pooled sensitivity and specificity of (18)F-FDG PET for detecting lung metastases across eight studies were 81% and 94%, respectively [26]. One study suggested that chest CT scans are more sensitive than PET scans for identifying pulmonary metastases from osteosarcoma. Additionally, it found that a negative PET scan cannot reliably rule out pulmonary metastases if suspicious findings are present on a chest CT scan [27].

**Figure 9:**
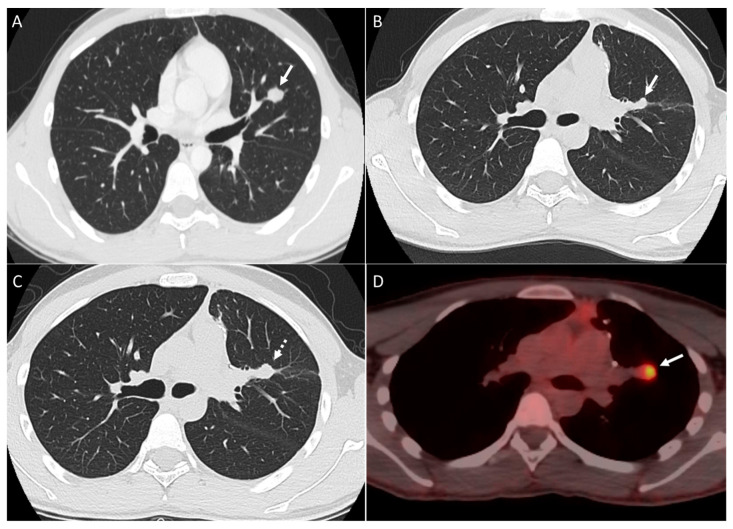
Patient with osteosarcoma and recurrent lung metastasis post metastasectomy. Axial enhanced image (**A**) shows a right upper lobe metastatic lung nodule (arrow). Axial non-enhanced image (**B**) status post metastasectomy from a follow-up CT shows nodular thickening along the surgical staple line (arrow). Axial non-enhanced (**C**) and axial fused PET CT (**D**) images show an interval increase in the size of the surgical staple line nodular thickening (dashed arrow) with FDG avid uptake (arrow) in keeping with disease recurrence.

**Figure 10:**
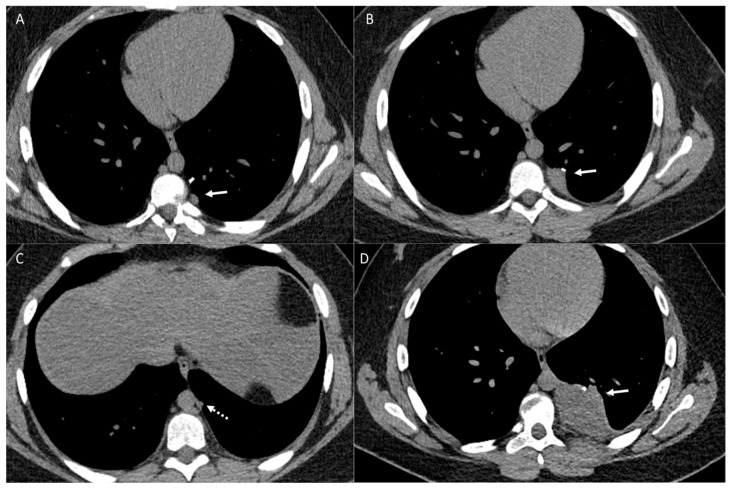
Patient with osteosarcoma and recurrent metastasis post metastasectomy. Axial non-enhanced image (**A**) shows a newly developed small left lower nodular pleural thickening near the surgical staple line (arrow). Axial non-enhanced images (**B**,**C**) show an interval further increase in the size of the previously seen nodular pleural thickening (arrow) and newly developed small one (dashed arrow). Axial non-enhanced image (**D**) shows a further significant increase in the size of the nodular thickening, becoming confluent and mass-like (arrow).

**Figure 11:**
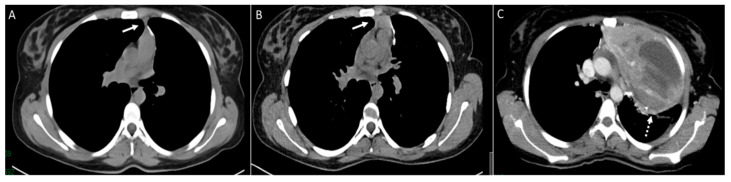
Patient with osteosarcoma and recurrent metastasis post metastasectomy. Axial non-enhanced image (**A**) shows post-operative changes along the surgical staple line (arrow). Axial non-enhanced image (**B**) from a follow-up exam shows interval development of mediastinal soft tissue thickening (arrow). Axial enhanced image (**C**) from a subsequent follow-up CT shows a marked interval enlargement (dashed arrow) representing mediastinal disease recurrence.

Pulmonary metastasectomy is always considered whenever feasible, as it is associated with prolonged survival. A retrospective analysis reported a 37% 5-year overall survival rate in patients who underwent thoracotomy, while none of the patients who did not undergo surgery survived [28]. The number, distribution (unilateral versus bilateral), and site (peripheral versus central) of metastatic lung nodules carry significant prognostic value, with centrally located lesions often requiring more invasive surgical approaches and associated with poorer outcomes [29,30,31]. CT surveillance post metastasectomy is crucial for detecting residual or recurrent disease. New or enlarging lung or pleural nodules, or nodular thickening along surgical scars and staple lines, should raise suspicions of recurrence (Figure 7, Figure 9, Figure 10 and Figure 11) [32].

## 3. Pleura

**Figure 12:**
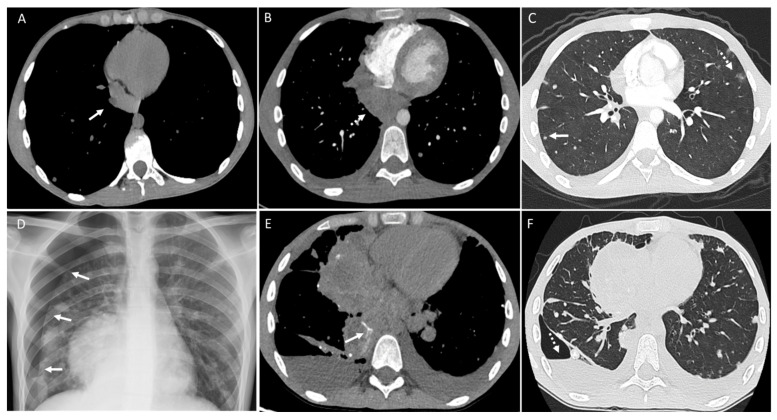
Patient with osteosarcoma and lung and pleural metastasis. Axial non-enhanced image (**A**) shows a right mediastinal pleura-based soft tissue lesion (arrow). Axial enhanced image (**B**) from a follow-up CT shows an interval increase in the size of the pleural-based soft tissue mass (dashed arrow) with a new small pleural effusion. Axial enhanced image (**C**) shows multiple lung nodules, including subpleural solid nodules (arrow), and nodules with a “halo sign” (dashed arrow). Frontal chest radiograph (**D**) from a 1-month follow-up shows interval development of right pneumothorax (arrows). Axial non-enhanced images (**E**,**F**) show a further increase in the size of the mediastinal pleura mass with interval development of intralesional calcification/ossification (arrow), left pleural effusion, and right hydropneumothorax (dashed arrow) in a subsequent follow-up exam.

**Figure 13:**
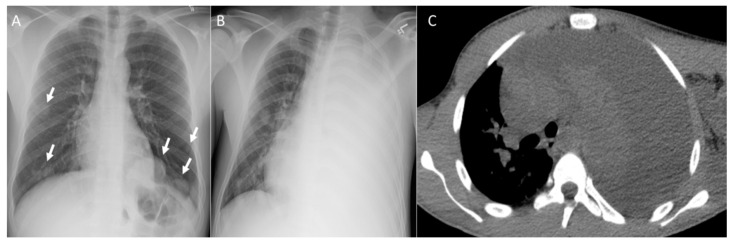
Patient with osteosarcoma with lung metastasis and malignant pleural effusion. Frontal chest radiograph (**A**) shows bilateral lung nodules and pleural-based left lower lobe masses (arrows). Frontal chest radiograph (**B**) and non-enhanced axial CT image (**C**) show an interval development of massive malignant left pleural effusion in a subsequent 3-week follow-up exam.

**Figure 14:**
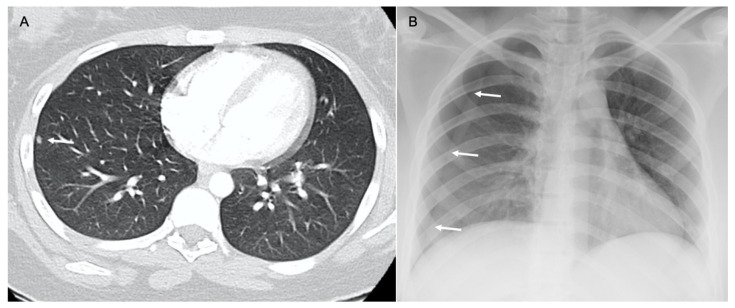
Patient with osteosarcoma and lung metastasis presented with spontaneous pneumothorax. Axial enhanced CT image (**A**) shows a right lower lobe subpleural lung nodule (arrow). Frontal chest radiograph (**B**) 5 days after the CT shows a medium-size right pneumothorax (arrows).

Pleural metastasis from osteosarcoma is uncommon and typically occurs alongside pulmonary metastasis, with isolated pleural disease being rare [12,33,34]. Pleural metastasis can result from direct extension of pulmonary metastases or via a hematogenous route [12]. Common CT findings include focal, multiple, or diffuse pleural nodules, nodular thickening, and pleural masses, often associated with concomitant pulmonary metastatic disease. Additional imaging features may include focal or diffuse dense pleural ossification and pleural effusion (Figure 10, Figure 12 and Figure 13) [11,12,34,35,36]. Although uncommon, spontaneous pneumothorax can occur in osteosarcoma patients with lung metastasis (Figure 14) [37]. This may be due to the transpleural rupture of cystic or necrotic metastases. Cases of spontaneous pneumothorax after chemotherapy initiation suggest that tumor cavitation induced by treatment could play a role [12,38]. Hemothorax has also been reported, likely related to tumor necrosis and transpleural rupture [39].

## 4. Mediastinum and Hilum

**Figure 15:**
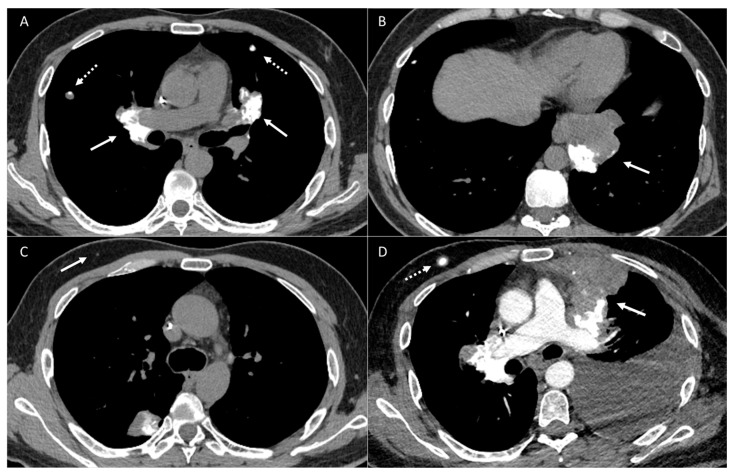
Patient with osteosarcoma multi-compartment thoracic metastasis. Axial non-enhanced image (**A**) shows bilateral calcified/ossified metastatic pulmonary nodules (arrows) and hilar lymphadenopathy (dashed arrows). Axial non-enhanced image (**B**) shows a left partially calcified/ossified pleural-based metastatic mass (arrow). Axial non-enhanced image (**C**) shows a small right anterior chest wall subcutaneous nodule (arrow) and a right lung subpleural nodule with calcification. Axial enhanced image (**D**) from a follow-up CT shows an interval increase in the size of the metastatic subcutaneous nodule with new calcification/ossification (dashed arrow), development of a left anterior pleural-based metastatic mass (arrow), and new left large left pleural effusion.

Lymphatic spread of osteosarcoma is uncommon, with fewer than 4% of patients presenting with clinically detectable lymph node metastasis at initial diagnosis [5,40]. Osteosarcoma metastasis to the chest can manifest as mediastinal and hilar lymphadenopathy (Figure 1 and Figure 15). The calcifications within metastatic lymphadenopathy can mimic granulomatous diseases such as tuberculosis. However, the absence of lymphadenopathy on baseline CT and the presence of other metastatic lesions in the chest typically allow for differentiation [11,12,41]. Extensive thoracic lymphadenopathy can lead to mass effect or invasion of adjacent cardiovascular structures, airways, and the esophagus [11,12,42].

## 5. Cardiac and Major Thoracic Vessels

Regional vascular invasion occurs in approximately 2% of resected extremity bone and soft tissue sarcomas and is associated with a poor prognosis [43]. Cardiac macroscopic thrombi from osteosarcoma have been reported in a limited number of cases, highlighting the rarity of this condition [18,44]. Imaging studies reveal that cardiovascular macroscopic metastasis from osteosarcoma is present in about 4% of cases, primarily in advanced disease [18]. Cardiac metastasis may result from hematogenous spread, direct extension from caval tumor thrombus into the right cardiac chambers, or from pulmonary vein tumor thrombus extending into the left cardiac chambers due to pulmonary metastasis [18,44].

**Figure 16:**
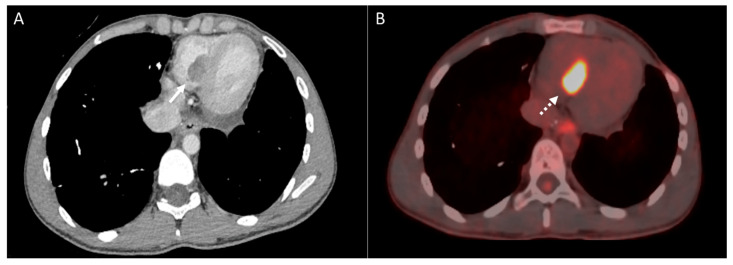
Patient with osteosarcoma and cardiac metastasis. Axial enhanced (**A**) and axial fused PET-CT (**B**) images show right ventricular tumor thrombus (arrow), which demonstrates FDG avid uptake (dashed arrow).

Distinguishing between tumor thrombus and bland thrombus on initial imaging can be challenging. Bland thrombus often improves or resolves on follow-up imaging, and veins affected by chronic bland thrombus typically decrease in caliber. In contrast, imaging features suggestive of tumor thrombus include heterogeneous enhancement on contrast-enhanced exams, pulsatile waveforms on Doppler images, and increased metabolic activity on (18)F-FDG PET (Figure 16) [18].

Dual-energy CT images are captured at low and high energy levels, typically around 80 and 140 kVp. By analyzing the attenuation data from these different X-ray spectra, material density and composition can be determined [45,46,47]. This method enables the differentiation of iodine-enhancing lesions, such as tumors, from non-enhancing thrombi [46,47]. Tumor thrombi, in particular, exhibit solid density and noticeable iodine uptake, characteristic of vital and vascularized tissues [47].

Peripheral pulmonary artery intravascular metastasis from osteosarcoma is increasingly recognized in clinical practice, largely due to improvements in CT technology and the availability of thin-slice CT images; this entity is discussed further in the section on pulmonary metastasis.

## 6. Chest Wall

**Figure 17:**
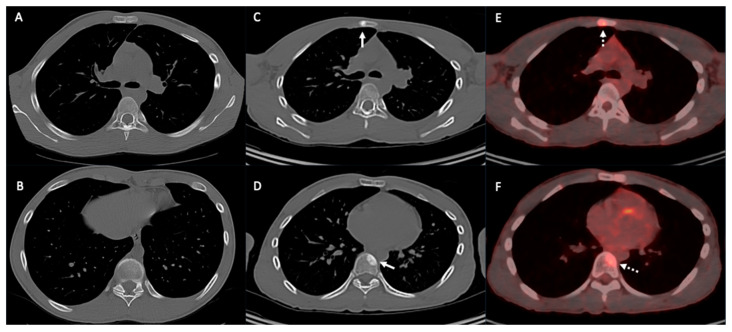
Patient with osteosarcoma and bone metastasis. Axial non-enhanced images (**A**,**B**) show no osseous lesions. Follow-up axial non-enhanced CT (**C**,**D**) and axial fused PET CT (**E**,**F**) images show an interval development of sternal and vertebral sclerotic lesions (arrows) with FDG avid uptake (dashed arrows) in keeping with metastasis.

Bone metastases from osteosarcoma can present at the onset of the disease. A retrospective review reported a 14% incidence of isolated bone metastasis at diagnosis [7]. Bone metastasis as the first sign of disease relapse is less common, occurring in about 10% of cases [48]. Multiple bone metastases are linked to poor prognosis, though it remains unclear whether these represent true metastases or multiple primary tumors [7,48]. (18)F-FDG PET can detect bone metastases even when CT findings are inconspicuous [49]. Osteosarcoma bone metastases may appear sclerotic or lytic with a sclerotic component (Figure 17) [12,49]. Distal soft tissue metastasis is rare, with only a few reported cases, but new or enlarging ossified nodules are highly suggestive of metastatic deposits (Figure 15) [50,51,52].

## 7. Role of Chest CT

Baseline and follow-up chest CT exams are crucial in managing osteosarcoma, as they enable early detection and timely treatment of metastatic disease [9,25,53]. While chest radiographs remain valuable postoperatively, especially for detecting complications like pleural effusion or pneumothorax after metastasectomy [9], chest CT demonstrated superiority over chest X-ray in detecting lung metastasis, particularly for small nodules. In patients with extremity osteosarcoma, a follow-up strategy using chest CT results in higher rates of second complete remission and better long-term outcomes, particularly in the first five years, compared to chest radiographs, due to its ability to detect recurrences early and guide effective surgical intervention [54].

Current guidelines recommend strict surveillance of both the primary tumor site and the thorax, particularly in the first 4–5 years. The follow-up schedule for chest CT after chemotherapy completion typically involves scans every 3 months for the first 2 years, every 6 months during years 3–5, and every 6–12 months from years 5–10, with subsequent intervals ranging from 0.5 to 2 years depending on clinical factors. Low-dose CT protocols are recommended, especially for younger patients, to minimize radiation exposure [53].

During follow-up, it is important to systematically evaluate the thoracic structures, including the lungs, mediastinum, pleura, chest wall, and cardiovascular structures. Pulmonary lesions should be carefully characterized in terms of size, number, and distribution, as the number of nodules on thin-slice CT at diagnosis may have greater prognostic value than nodule size [24]. The use of maximum-intensity projection (MIP) and reduced slice thickness can significantly improve the detection of small nodules (<5 mm) [55,56].

The Response Evaluation Criteria in Solid Tumors (RECIST) guidelines are commonly used for measuring tumor response during follow-up [57]. However, RECIST has limitations, particularly for osteosarcoma, where small, multiple pulmonary nodules may be clinically significant [58].

The standard chest CT protocol includes single-breath-hold volumetric acquisition from the thoracic inlet to the diaphragm, with or without intravenous contrast. Intravenous contrast may be useful in cases with suspected involvement of hilar or mediastinal structures.

## 8. Conclusions

Osteosarcoma metastasis to the thorax is a common and significant contributor to morbidity and mortality. While lung nodules are the most frequent manifestation, metastatic disease can involve a wide range of thoracic structures, including the pleura, vasculature, lymph nodes, and chest wall. Recognizing the spectrum of typical and atypical radiographic patterns across all thoracic compartments is essential for timely diagnosis, effective treatment, and improved survival outcomes.

## Abbreviations

Computed tomography (CT), (18)F-fluorodeoxyglucose positron emission tomography [(18)F-FDG PET], Response Evaluation Criteria in Solid Tumors (RECIST).
